# Complement System in Pathogenesis of AMD: Dual Player in Degeneration and Protection of Retinal Tissue

**DOI:** 10.1155/2014/483960

**Published:** 2014-09-04

**Authors:** Milosz P. Kawa, Anna Machalinska, Dorota Roginska, Boguslaw Machalinski

**Affiliations:** ^1^Department of General Pathology, Pomeranian Medical University, Al. Powstancow Wlkp. 72, 70-111 Szczecin, Poland; ^2^Department of Ophthalmology, Pomeranian Medical University, Al. Powstancow Wlkp. 72, 70-111 Szczecin, Poland; ^3^Department of Histology and Embryology, Pomeranian Medical University, Al. Powstancow Wlkp. 72, 70-111 Szczecin, Poland

## Abstract

Age-related macular degeneration (AMD) is the most common cause of blindness among the elderly, especially in Western countries. Although the prevalence, risk factors, and clinical course of the disease are well described, its pathogenesis is not entirely elucidated. AMD is associated with a variety of biochemical abnormalities, including complement components deposition in the retinal pigment epithelium-Bruch's membrane-choriocapillaris complex. Although the complement system (CS) is increasingly recognized as mediating important roles in retinal biology, its particular role in AMD pathogenesis has not been precisely defined. Unrestricted activation of the CS following injury may directly damage retinal tissue and recruit immune cells to the vicinity of active complement cascades, therefore detrimentally causing bystander damage to surrounding cells and tissues. On the other hand, recent evidence supports the notion that an active complement pathway is a necessity for the normal maintenance of the neurosensory retina. In this scenario, complement activation appears to have beneficial effect as it promotes cell survival and tissue remodeling by facilitating the rapid removal of dying cells and resulting cellular debris, thus demonstrating anti-inflammatory and neuroprotective activities. In this review, we discuss both the beneficial and detrimental roles of CS in degenerative retina, focusing on the diverse aspects of CS functions that may promote or inhibit macular disease.

## 1. Introduction

Age-related macular degeneration (AMD) is the leading cause of irreversible visual impairment and disability among the elderly worldwide, and AMD currently affects almost two-thirds of the population over 80 years old [1, 2]. Moreover, AMD is the main cause of blindness, and 10–18% of individuals between 65 and 75 have lost some central vision as a result of AMD [3]. Moreover, the prevalence of this condition is predicted to rise as the elderly population expands. Indeed, in the USA, it was estimated that a 50% increase in the number of affected individuals will be observed between 2004 and 2020 [1].

The two major types of AMD are exudative or neovascular (“wet”) form and nonexudative (“dry”) AMD. Although most patients with AMD suffer from the dry, nonexudative form of the disease, approximately 10–15% of the AMD cases are the exudative type. The earliest clinical manifestation and pathological feature of AMD is the development of extracellular deposits called drusen located inside Bruch's membrane (BM) and beneath the retinal pigment epithelium (RPE). A few small drusen can be found in healthy individuals over age 50, but the presence of large or numerous drusen is a risk for AMD [4]. The early stage of dry AMD is also characterized by pigmentary abnormalities in the macula. In more advanced stages of dry AMD, patients experience a loss in RPE and thinning of the photoreceptor layer in the macula, leading to retinal atrophy and surrounding tissue death in a process known as geographic atrophy (GA) [5]. The “wet” form of AMD is characterized by choroidal neovascularization (CNV), which is the growth of abnormal blood vessels from the choroid underneath the macula. The abnormal blood vessels eventually result in a disciform scar, leading to permanent loss of central vision [5]. Despite intensive basic and clinical research, the pathogenesis of AMD remains unclear. However, a growing body of evidence indicates that AMD is likely a multifactorial, progressive disease that involves complex interactions between genetic and environmental factors [6]. It is widely accepted that inflammatory and immunologic events might play a role in AMD. During the last decade, convincing evidence has emerged implicating the complement system (CS) in AMD pathogenesis [7]. Initially, several CS factors, their activators, and complement regulatory proteins were identified as cardinal constituents of drusen, the hallmark extracellular retinal deposits associated with early AMD [8]. Moreover, genetic studies revealed statistically significant associations between AMD and polymorphisms of several complement pathway-associated genes [9]. Finally, the abnormal concentrations of primary complement factors and their activated products have been observed in the circulation of AMD patients [10]. Nevertheless, ultimate studies also demonstrate that the absence of complement components is deleterious to retinal homeostasis. Thus, CS is increasingly recognized as mediating an important role in retinal biology.

In this review, a short summary of the function of the complement system in retinal tissue will be followed by a discussion on the positive and detrimental roles of complement system in degenerative retina that considers how the diverse functions of CS may promote or inhibit AMD disease.

## 2. Biology of Complement System in Retina

The complement system, an integral part of the humoral immune system, is involved in tissue inflammation, cell opsonization, and cytolysis, not only protecting against microorganisms but also mediating the clearance of exogenous and endogenous cellular debris from the host tissues. Therefore, CS functionality is extremely important for the maintenance of cellular integrity, tissue homeostasis, and temporal modifications of the adaptive immune responses of the organism [11].

**Figure 1:**
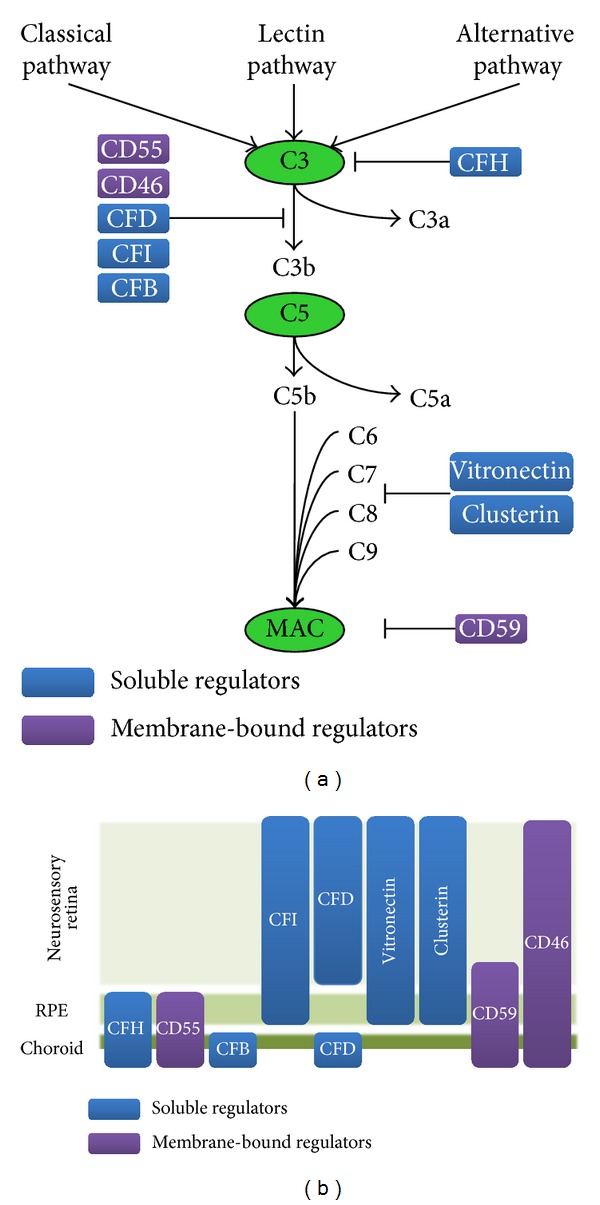
(a) Complement system activation. Local effects of the inhibitors on complement components. (b) Complement system in the eye. Local production of the complement inhibitors by retinal cells.

The CS cascade is comprised of four activation pathways. All of the pathways ultimately end in the central cleavage of C3 factor and in the generation of its active fragments C3a and C3b. C3a is the anaphylatoxin that triggers a range of chemotactic and proinflammatory responses, such as recruitment of inflammatory cells and increased microvasculature permeability, whereas C3b is responsible for opsonization of foreign surfaces covalently attached to C3b (see Figure 1). Opsonization with activated C3 fragments (C3b and iC3b) fulfils three major functions: (i) cell debris elimination by phagocytic cells (e.g., macrophages or microglia) and the stimulation of the adaptive immune system (B and T cells); (ii) amplification of complement activation via the formation of a surface-bound C3 convertase; and (iii) assemblage of the C5 convertase. The latter event is responsible for C5 cleavage, which results in the formation of the cytolytic membrane attack complex (MAC) capable of generating perforations in the cell membrane, thereby promoting cell lysis and the elimination of unnecessary cells [12]. Through all of these activities, the innate complement cascade supports and promotes the function of downstream mechanisms of the immune system that protect the integrity of the host tissue. Overall, CS pathway activation results in a proinflammatory response, including MAC generation, which mediates cell lysis, the release of chemokines to attract inflammatory cells to the site of damage, and the enhancement of capillary permeability to promote extravasation of infiltrating leukocytes. Under physiological conditions, complement activation is effectively controlled by the coordinated action of soluble and membrane-associated complement regulatory molecules (CRMs). Soluble complement regulators, such as C1-inhibitor, anaphylatoxins inhibitor, C4b binding protein (C4BP), complement factor H (CFH), complement factor I (CFI), clusterin, and vitronectin, restrict the action of complement in human tissues at multiple sites of the cascade reaction (see Figure 1). In addition, each individual cell is protected against the attack of homologous complement by surface proteins, such as the complement receptor 1 (CR1, CD35), the membrane cofactor protein (CD46), and glycosylphosphatidylinositol-anchored proteins, such as decay-accelerating factor (CD55) or CD59 molecule [12]. Of note, host cells and tissues that are inadequately protected from complement attack might be subjected to bystander cell lysis [13].

In recent times, the identification and characterization of the local complement system in the RPE-choroid-neurosensory retina complex empowered our knowledge regarding the biological complexity of the role of the complement system in ocular tissues, specifically in the retina. Identification of the complement-related protein profile of different parts of the eye permits distinguishing the physiological and pathological features of the ocular tissues in case of disease development. The main components of the complement system, including C3, C5, and the MAC complex, are normally present in the capillary vessels of the choroid and the vitreous of human eyes [14–17]. Interestingly, Anderson et al. revealed that the predominant cellular source(s) for most classical and alternative pathway components, including the shared C3 factor, reside(s) in the choroid rather than in the RPE or neural retina [13]. In addition, they also performed a detailed analysis of the localization in the neurosensory retina, RPE, and choroid of several CRMs important for prevention of excessive complement activation [13]. Accordingly, it was found that CFH immunolabelling was confined primarily to the choriocapillaris and intercapillary pillars and CD55 immunoreactivity was limited to the inner aspect of choroidal capillaries. In addition, CFB immunolabelling was localized throughout the choroid and was also associated with the luminal surfaces of choroidal endothelial cells. In contrast, CFI labelling was highest in the inner retina and relatively low in the choroid, and CFD labelling appeared to be diffusely distributed throughout the entire neural retina and choroid [13]. With regard to the three terminal pathway inhibitors (clusterin, vitronectin, and CD59), their expression levels were similar in the choroid and RPE; however, the neural retina displayed comparable levels of both clusterin and vitronectin but little detectable CD59 [13]. Anderson et al. also determined that the membrane-bound complement regulatory molecule CD46, which is an inhibitor of the alternative pathway, displays the robust levels of transcription in the choroid, RPE, and neural retina [13]. In addition, intense CD46 protein immunoreactivity was observed along the basolateral surface of the RPE cells and, to a lesser extent, on the luminal surface of choroidal endothelial cells [13]. The authors of this complex and detailed study concluded that CS activators and inhibitors are abundantly expressed by resident cells of the choroid tissue, whereas the presence of CS activators and inhibitors in both neural retina and RPE is limited to a subset of particular inhibitors of the alternative (CD46, CD55, CFH, and CFI) and terminal (clusterin, vitronectin, and CD59) pathways. In summary, the characteristic profile of retinal expression of selected CRMs may be relevant in regulating local inflammatory processes in different parts of retinal tissue. Moreover, the pathological age-related changes in the expression of selected CRMs, especially in neural retina and RPE, may predispose to complement-mediated inflammation, and it might be an important factor for AMD pathogenesis.

In this notion, a great body of evidence suggests that complement activation is augmented in retinal tissue with age. In vivo, the increased expression of nine different CS factors was observed in RPE/choroid complexes collected from aged mice [18]. Moreover, the tissue localization of C3 was different in young and old animals, revealing possible disturbances in the functional integrity of RPE/BM complex as a result of aging [18]. Likewise, upon analysis of the transcriptome of human RPE collected from aged healthy subjects, it was found that the RPE highly expresses genes of the complement cascade (e.g., C3, CFB, CFH, HTRA1, CST3, and FBLN5) [19]. It was also observed that basal CS activation in cultured RPE cells increases steadily with age [20]. Concurrently, this activation stimulates the age-induced response of augmented synthesis of endogenous CRMs, such as CFH [21, 22] or CFB [20]. Moreover, Kociok and Joussen observed that human RPE cells that are stimulated to proliferate in culture conditions express CRMs, such as CFH and CFI, at the mRNA and protein levels [23]. Interestingly, in cultured human and murine RPE cells, the membrane-bound CRMs, such as CD46, CD55, and CD59, were also shown to be specifically upregulated by inflammatory cytokines, including IFN-*γ*, TNF-*α*, and IL-1*β*, as well as by repetitive nonlethal exposure to oxidative stress [24]. This phenomenon seems to be especially important as protective mechanism in the natural aging of the retina as well as in retinal age-related diseases. CD46 immunoreactivity has also been localized to RPE cells overlying retinal drusen [16]. Moreover, analysis of expression levels of various CRMs indicated that CD59a mRNA and protein were more abundant in the murine eyecups than in nonocular tissues, suggesting that CD59a may play an important role in protecting the mouse posterior segment from complement-mediated injury [24]. Similarly, the importance of retinal expression of CD59a has also been reported in the mouse model of laser-induced choroidal neovascularization [25]. Interestingly, recombinant CFH protein induced dose-dependent chemotactic migration of human RPE cells towards the increasing CFH concentration gradient [22]. Overall, the upregulation of complement inhibitors in retinal tissue observed in vivo and in vitro may indicate the body's natural attempt to restore biological balance to the augmented immune responses in physiological mechanisms, such as aging.

However, several of the regulatory mechanisms described above may be overwhelmed when the CS is improperly activated, resulting in tissue destruction and degenerative disease processes, such as AMD. Importantly, the pathological effects of CS are mediated by precisely the same mediators responsible for the CS's well-known protective roles. Therefore, we hypothesize that different CS components play dual roles, that is, positive, if they protect the tissue against injury, and negative, if they produce tissue damage due to local inflammatory reactions mediated by activated CS components and their products. For example, the MAC complex is responsible for proteolysis of cellular debris, but it may injure other living cells if increased amounts of MAC have been deposited in a particular tissue. On the other hand, the deposition of MAC at sublytic concentrations has been associated with the inhibition of apoptosis through enhanced synthesis of bcl-2 and inhibition of caspase-3 activation [26]. Low concentrations of MAC also induce prosurvival signals in Schwann cells through phosphatidylinositol-3-kinase-mediated phosphorylation of BAD or cell cycle activation in oligodendrocytes [27, 28].

In conclusion, if unregulated, CS activation may directly damage host tissue and recruit immune cells to the vicinity of active complement cascade. Therefore, the protection against complement is achieved through various CRMs that are widely expressed in and secreted to the retina. In particular, the choroid- and RPE-based regulation of the complement system activity play an important role in the eye. Of note, these retinal elements are also strongly implicated in the pathogenesis of AMD.

## 3. Genetic Mutations in CS Component Genes and Their Dual Role in AMD Pathogenesis

AMD, a complex genetic disorder, is attributed to multiple genes and their modifications [29]. Single nucleotide polymorphic changes are considered normal variants of gene structure, which may protect or predispose to various conditions, including AMD. The studies performed in American twins revealed that the heritability of AMD, that is, the relative contribution of genetic and nongenetic factors to the phenotype, is estimated at a relatively high value of between 45% and 70% [30]. It was also estimated that currently identified loci account for approximately 55% of AMD heritability [31]. Recently, the international scientific group performed the multicenter meta-analysis involving more than 17 000 cases of AMD and 60 000 control patients defining an association between AMD and genetic variants in a total of 19 chromosomal regions [32]. Of note, it was observed that the same polymorphisms that are considered to be involved in the pathogenesis of early AMD do not appear to influence the rate of progression of advanced AMD, including CNV or GA [33]. Thus, the expression of certain genes may reveal both protective and detrimental characteristics, depending on the phase of the disease (see Table 1).

Following the discovery that retinal drusen contain CS-related proteins, several reports appeared in 2005 and stated that the chromosomal region 1q31 that encodes CFH is a major susceptibility locus for AMD according to whole-genome association analyses performed independently in three different cohorts [45–47]. The CFHY402H allele (rs1061170, T > C) in exon 9 of CFH confers a significantly increased risk of AMD. Individuals with the risk allele “C” exhibited an increased risk of AMD with an odds ratio (OR) of 2.4–4.6 for a single copy and 3.3–7.4 for two copies of the variant allele. In general, this risk haplotype fails to recruit the inhibitory protein CFH to the sites where complement is activated by the accumulation of endogenous complement-activating compounds. Recently, the R1210C CFH mutation has also been found to be associated with the early onset of AMD [55]. Similarly, five different polymorphisms in the complement factor H-related gene 5 (CFHR5) that encodes the inhibitory protein for C3b have been described and associated with an increased risk of AMD [56]. Altogether, the complex gene family composed of CFH and CFHRs genes appears to be a genetic hotspot, where various genetic rearrangements have strong functional implications and result in the different disease-associated polymorphisms, including AMD. This observation is attributed to the fact that the CFH and FHR proteins encoded by CFH and CFHRs genes closely collaborate to control complement activity in different cells and their deregulation induces tissue degeneration. In the following years, the selected candidate gene studies have also identified associations of complement-related genes with AMD development and progression, including different variants of complement factor B (CFB32Q rs641153), factor C3 (C3102G rs2230199), factor CFI (rs4698775), or factors C4A or C4B, which both flank rs429608 locus [32, 38, 57–59]. Till now, the most consistent and significant association with AMD risk was found in case of SNP rs10490924 located in chromosome 10q26, in the region of ARMS2 and HTRA1 genes [32]. This risk variant is associated with a twofold increase in expressed HTRA1 protein [60], which activates both the alternative and classical complement pathways through elimination of several inhibitory CRMs.

On the other hand, the protective haplotypes have also been identified in some complement-related genes. Likewise, several studies provided a strong evidence for a protective role of selected haplotypes of complement factor H (CFH) characterized by a deletion of two additional members of the CFH gene family, such as the complement factor H-related genes 1 and 3 (CFHR1 and CFHR3) [53, 61]. These two proteins share binding properties with CFH through significant amino acid sequence homology. Moreover, CFHR1 appears to act downstream by modulating the activity of the C5 convertase and inhibiting MAC formation. Therefore, it has been proposed that the protective effect in AMD conferred by the deletion of CFHR1/CFHR3 is mediated by removal of the C5a blockade and disinhibition of MAC formation [62]. This advantageous scenario potentially results in enhanced clearance of the cellular debris and drusen, which, if present, naturally promote the chronic local inflammatory state and subsequent atrophy of RPE in the macular area. This molecular finding suggests that the binding of various CFH-related FHR proteins and their capacity to regulate complement activity, directly or by competing with CFH, can be beneficial for neurosensory retina [63]. Of note, this polymorphism is especially common in Caucasians [53]. Additionally, the other CFH coding variant rs800292 (I62V) is also protective against AMD. This SNP is located in the second exon of CFH and results in the substitution of an isoleucine (I) amino acid instead of a valine (V) in the first domain of the protein [49]. Furthermore, the polymorphisms in other complement-related genes are also associated with decreased susceptibility to AMD [34, 37, 64]. The good examples are complement factor B (CFB) and complement factor 2 (C2). Although both of them aid in initiation of the alternative and the classical CS pathway, respectively, some specific haplotypes or individual SNPs in these genes showed a protective effect against AMD [36]. Spencer et al. found that the R32Q variant of CFB was significantly associated with protection from AMD in the analyzed family-based data set [37]. Additionally, the same group found that three SNPs in C2 and CFB (C2 E318D; C2 rs547154; and CFB R32Q) were strongly associated with decreased risk of AMD in the analyzed case-control data set [37]. Accordingly, a significant protective effect of genotype CT/TT in CFB R32Q variant (rs641153) for GA and a positive trend for reduced risk of CNV were observed when compared with CC genotype [65]. Similarly, an association of the CG/CC genotype (versus GG) in C2 E318D variant (rs9332739) with protective effect against AMD and GA development was reported [65]. Thus, several risk and protective haplotypes in the complement-related genes modify susceptibility to AMD; however, the detailed mechanisms by which different CS gene polymorphisms impact AMD risk are not well known. Moreover, numerous polymorphisms may act collectively, leading to local CS dysregulation resulting in pathological features in retinal tissue. Thus, the performed genetic studies on selected cohorts of AMD patients revealed that multiple genetic mutations are often required to induce the specific disease phenotype. Nevertheless, the knowledge on the contribution of particular loci to AMD genetic susceptibility is essential for understanding the potential biological mechanisms through which these variations modulate the development this complex retinal disease.

Interestingly, several genetically determined haplotypes identified in AMD have specific patterns of complement activation and are associated with different plasma levels of individual complement components. For example, Hecker et al. found that the “high risk” and “neutral risk” CFH locus haplotypes did not alter plasma levels of CS proteins, whereas the protective haplotype (CFH402Y) decreases levels of Ba and C3d components. Similarly, the common “risk” haplotype spanning C2/CFB increased C3d levels, whereas the less common protective haplotypes decreased Ba and C3d levels. Finally, the nonsynonymous SNP that is related to the “high risk” C3 haplotype displayed increased C3d levels [66]. Moreover, the extensive study of Scholl et al. on systemic CS activation in AMD patients showed that complement hyperactivation in the peripheral circulating blood provided increased prognostic predictive power compared with the “simple” genotypic analysis of the same patients [67].

In conclusion, different complement-related genes and their variations displayed significantly strong associations with AMD progression or a reduced risk of AMD development. Regarding the above findings, the complexities associated with AMD genetics offer a challenging problem for understanding the molecular basis of AMD and its subsequent management in patients.

## 4. The Detrimental Role of the Complement System in Retinal Degeneration

The complement system has been implicated in early AMD pathogenesis based on the identification of CS components in drusen from eyes of AMD patients [8]. Drusen are the focal deposits of extracellular debris located between the basal lamina of the RPE and Bruch's membrane (BM). Major drusen constituents include cellular debris, lipids, proteins, and lipoproteins [68–70]. The structure and type of drusen significantly affect the rate of AMD development and progression [71]. In the last decade, multiple levels of significance have been ascribed to the molecules trapped or sequestered in drusen, including toxicity to the overlying RPE, extracellular enzymatic processing, impaired transport of molecules across the BM, induced extraretinal cellular invasion, and effectors of the pathological process affecting RPE survival and metabolism. In AMD, at least 129 types of drusen-deposited proteins have been identified, including different apolipoprotein types (E, B, or A-I), several amyloid peptides (P, A*β*, or SA-1), TIMP-3, serum albumin, and certain proteins associated with cellular function (e.g., ATP synthase *β* subunit, scavenger receptor B2, and retinol dehydrogenase) [69, 72]. Most importantly, AMD-derived drusen also contain almost all of the complement proteins, including regulatory proteins (CFH, vitronectin, and clusterin), the products of CS activation and degradation (C1q, C3, C3a, C3b, and C5a), and members of the terminal CS pathway comprising the MAC components (i.e., 5, 6, 8 (*α*, *β*, and *γ*), and 9) in the separated and complex form [14, 69]. Thus, one of the main hypotheses indicates that the accumulating drusen may activate the CS, trigger the local production of inflammatory mediators, and attract leukocytes that in turn augment the local inflammatory state. Johnson et al. used a specific cell culture model of RPE cells to determine that exposure to human serum results in CS activation. This process is mediated by the classical CS pathway via binding of C1q to ligands in apolipoprotein E-rich deposits, thus triggering direct activation of complement by C1q with subsequent inflammatory sequelae [73]. Similarly, it was found that A*β* deposits accumulated in drusen and colocalised with activated complement components [74]. Indeed, more than a decade ago, Johnson et al. reported that the A*β* is a potential activator of CS and contributes to atrophy of the retinal pigmented epithelium, drusen biogenesis, and AMD pathogenesis [75]. Interestingly, C1q may have a potential role in the mechanism of drusen aggregation in the larger complexes, thus causing mechanical insult to the RPE as observed in AMD-related GA [76]. Moreover, the direct treatment of ARPE-19 cells with hydrogen peroxide, which strongly induces oxidative stress in vitro, reduces cell surface content of complement inhibitors, such as CD46, CD55, and CD59 [77]. This finding suggests that RPE cells exposed to oxidative insult allow for complement activation on their surfaces. Likewise, it was found that the CS components localized in drusen could induce inflammasome activation, thereby potentially promoting the negative cellular effects in the affected retinas [78]. For example, Doyle et al. found that C1q could activate the NLRP3 inflammasome locally in the retinas and activate tissue macrophages [78]. These actions subsequently rupture lysosomes and release the proteolytic lysosomal content to the extracellular matrix, thereby damaging the surrounding normal cells [79].

Likewise, several studies have also identified the complement system as an important component of laser-induced CNV development [25, 80–83]. In particular, MAC, the final product of the activated complement cascade, induces the release of growth factors, such as *β*-fibroblast growth factor (*β*-FGF), vascular endothelial growth factor (VEGF), and platelet-derived growth factor (PDGF), from various nucleated cells [80, 84]. Bora et al. observed that systemic pharmacological blockade of the entire complement system as well as the selective complete depletion of C3 using C3−/− knockout mice reduced the development of CNV (from 98% in control mice to 3–5% in C3−/− mice) [80]. The same group also observed strong MAC deposition in the CNV complex exclusively in wild-type control mice; in contrast, no MAC staining in the laser spots was observed in whole complement-depleted mice or in C3−/− mice. Similarly, mice treated with an anti-C6 antibody that blocks the generation of MAC complex displayed no visible MAC complexes and a significantly reduced CNV process [80]. Moreover, Bora and coworkers validated the neovascular role for MAC in CNV formation in an in vivo study and revealed significant inhibition of CNV formation in C5-deficient mice (C5−/−) [81]. Taken together, these studies support a detrimental role of MAC in CNV development. In addition, Nozaki et al. found that C3a and C5a induce VEGF expression in the mouse RPE in vitro and in vivo and thus may accelerate the model of neovascular AMD [82]. Similarly, Cashman et al. observed that the conditionally increased expression of C3 in RPE obtained by intraocular injection of an adenovirus-expressing murine C3 caused significant functional and anatomic changes in the retina, which reproduce many of the features characteristic of AMD and other retinal diseases [85]. In particular, exogenous expression of C3 induces both blood vessel leakiness and endothelial cell proliferation in the murine retina. Finally, the C3-injected eyes displayed significantly reduced cone and rod function as measured by ERG, and this reduction was consistent with the loss of photoreceptor segments [85].

Altogether, a growing body of evidence suggests that the CS pathways play a key role in the development of AMD. Complement system dysregulation in retina can lead to cell and tissue damage, which provokes the development of drusen acting as foci of chronic inflammation and subsequent CS activation. Likewise, chronic inflammatory responses and CS activation play an unquestionable role in CNV development and VEGF secretion.

## 5. The Protective Role of CS against Retinal Degeneration

Inflammation is often recalled in a negative light; however, the inflammatory processes have evolved as an initial response to restore homeostasis after cellular injury, that is, to protect and promote a return to the “status quo ante.” Therefore, the initial onset of inflammation is primarily beneficial to the host [86]. In this condition, complement activation in retinal drusen appears to be a double-edged sword; it can exacerbate tissue damage or promote cell survival and tissue remodeling by facilitating the rapid removal of dying cells and resulting cellular debris [86, 87]. Several CS components possess anti-inflammatory functions. Pisalyaput and Tenner have recently reported that C1q, in the absence of other complement components, increases neuronal survival and neurite outgrowth compared with untreated neurons and protects against *β*-amyloid-induced neurotoxicity in vitro [88]. These researchers also identified the molecular mechanisms underlying the observed neuroprotective effects of C1q using microarray analysis [88]. Benoit and Tenner found that C1q upregulates the expression of genes associated with neurite outgrowth (STX3) and downregulates the expression of genes associated with inflammation as well as microRNAs, including let-7c, microRNA-410, and microRNA-497, that specifically inhibit the expression of neurotrophic factors, such as nerve growth factor (NGF) and neurotrophin-3 (NT-3) [89]. Thus, C1q, expressed by neurons in response to neurodegeneration or injury, may play a protective role by enhancing the clearance of apoptotic cells and providing direct protective effects on neurons. The demonstrated C1q-dependent neuroprotection suggests that C1q can induce a potent and novel neuroprotective mechanism in injured neurons.

One potential neuroprotective activity of CS is indicated by the results of experiments performed on C3-deficient (C3−/−) animals. Maier et al. found that complete C3 deficiency leads to accelerated amyloid *β* plaque deposition with animal aging and thus augments neurodegeneration [90]. The authors conclude that the presence of C3 is potentially necessary for anti-inflammatory cytokine-mediated stimulated microglial phagocytosis of amyloid and other cellular debris. Interestingly, recently published results indicate that deficiencies in the CS components (such as C3 or CFH) are deleterious to retinal physiologic state and function. Kam et al. examined retinal function in 12-month-old (C3−/−) mice compared with mice deficient in both C3 and CFH (CFH−/−C3−/−), only CFH (CFH−/−), and control wild-type mice [91]. Although all of the analyzed mutant mice displayed significant photoreceptor loss and thickening of Bruch's membrane compared with control mice, the C3−/− strain presented significantly more A*β* in Bruch's membrane, a reduced number of macrophages, and increased levels of retinal inflammation, including increased expression of inflammatory markers: TNF-alpha and calcitonin, compared with the other examined mice [91]. Importantly, the inflammatory process identified in the retinas of C3−/− mice was even higher than that observed in CFH−/− mice, which normally display uncontrolled C3 activation through the alternative CS pathway given the complete CFH deficiency. Finally, the scanning electron microscope analysis revealed the excessive accumulation of fibrillary debris material on the photoreceptor outer segments. Interestingly, abnormalities within neural retina (e.g., reduced electrophysiologic response, reduced photoreceptor number) were significantly increased in mice with a combined deficiency of CFH and C3 than compared with mice with CFH or C3 deficiency alone [91]. These data indicate that both CFH and C3 are required to maintain retinal physiological function and that active C3 protein mediates an important role in retinal biology.

It is widely accepted that complement inhibition through C3aR blockade may be an effective mechanism for preventing or slowing AMD progression [92, 93]. However, various reports have demonstrated in vitro that C3a may act as an anti-inflammatory molecule by suppressing LPS-induced secretion of TNF-*α*, IL-1*β*, and IL-6 from human peripheral blood mononuclear cells and stimulated B lymphocytes as well as upregulate anti-inflammatory cytokine gene expression [94, 95]. Likewise, in nervous tissue, C3a induces substantial neuroprotective effects by inhibiting excitotoxicity-mediated neuronal death through astrocyte stimulation in mixed cultures of neurons and astrocytes [96, 97] or stimulating microglia to produce neurotrophic growth factors [98]. Several groups postulate the C3a-dependent stimulation of microglial secretion of neurotrophins, such as NGF, that are involved in early stages of neuronal regeneration [97, 98]. C5a exposure also causes an upregulation of NGF mRNA expression in astrocytes [97]. Administration of C5a in vivo was also reported to protect against kainic acid-induced neuronal apoptosis [99].

With regard to this notion, evidence suggests that C3aR and C5aR-induced signaling may be important for the normal retina to maintain its structure and function and may be involved in retinal repair and regeneration. C3aR and C5aR are the members of the class A subfamily 8 of G protein-coupled receptors (GPCRs), and GPCRs signaling is highly important for cell growth, cell activity, and survival. Although the GPCR signaling can vary across different cell types, the general physiologic outcome has prosurvival and antiapoptotic functions. Indeed, the activation of C5aR in macrophages, dendritic cells, and neurons induces several important prosurvival pathways, such as MAPK, PI3K-Akt, NF-*κ*B, and cAMP response element-binding signaling [100–102]. Moreover, active C3aR induces the NF-*κ*B-dependent pathway in tubular epithelial cells [103] and transcription factors activator protein-1 in glial cells [104]. Moreover, both receptors activate the PI3K-Akt kinase pathway in T cells [105]. Yu et al. recently reported that mice deficient in receptors for anaphylatoxins (C3aR−/− and/or C5aR−/−) presented abnormalities similar to C3−/− mice, and they developed progressive retinal degeneration and retinal dysfunction [106]. These mice showed a significantly greater loss in retinal function as measured by ERG. For example, the ERG a- and b-waves of C3aR−/− and C3aR−/−C5aR−/− mice were significantly reduced in 14-week-old animals, and this reduction progressed with increasing of age. Furthermore, a progressive retinal cell loss of photoreceptors, bipolar cells, and horizontal cells was observed in C3aR−/− mice. In particular, the number of rod bipolar cells was significantly decreased at 3 months and declined further at 14 months of age. It was proposed that apoptosis activation is primarily responsible for the retinal cell number decline as increased expression of activated caspase-3 and decreased NF-*κ*B activation were observed in retinal cells of C3aR−/− and C3aR−/−C5aR−/− mice. Morphologic analysis by electron microscopy revealed that mice lacking C5aR or C3aR display shrunken RPE cell nuclei, clearly indicating intracellular RPE dysfunction in these mice. In addition, the normal structure of outer segment discs was lost in 6-month-old C3aR−/−C5aR−/− mice. Finally, based on performed biochemical studies, the authors indicate that C3aR may play an important role in protecting retinal cells from light-induced retinal degeneration through activation of the NF-*κ*B pathway [106].

In light of the above findings, it is worth mentioning that the neuroprotective functions of C3a and C5a include protection against NMDA- [96] and glutamate-induced apoptosis [99] via MAPK-dependent inhibition of caspase 3 [107] and regulation of glutamate receptor subunit-2 [108]. This finding is an especially important concern in light of all the available data suggesting that mice lacking C3a and/or C5a signaling also display several abnormalities in nervous system and internal organ function, for example, anomalous susceptibility to neuroexcitotoxicity [109], abnormal differentiation and migration of neural progenitor cells [110], and abnormal neuron remyelination [111]. With regard to the role of anaphylatoxins in neurogenesis, the receptors for C3a and C5a (C3aR and C5aR, resp.) are expressed on neural progenitor cells and immature neurons [112]. Mice treated with a nonspecific C3aR antagonist (SB290157) displayed decreased formation of new neurons in areas of adult neurogenesis [112].

Altogether, the above results strongly indicate a continuous necessity for the active complement pathway in the normal maintenance of the neurosensory retina and robustly support the notion that disturbances in the physiological activation of CS may have detrimental effects for retinal biology. Although deregulation or excessive activation of CS following neurodegenerative events has emerged as a major contributor to secondary tissue damage, numerous findings suggest that regular complement activation is a necessary part of normal physiological function of nervous tissue, including retinal cells.

## 6. Conclusions

CS activation may possess a dual role in retinal structure and function as well as in the pathophysiology of retinal degenerative diseases, such as AMD. To the best of our knowledge, this is the first comprehensive informative study that describes thoroughly the topic of dual role of CS in degeneration and protection of retinal tissue. On the one hand, the CS can have beneficial effects by facilitating phagocytosis and the removal of cellular debris and drusen components; however, the CS can also be detrimental by causing bystander damage to surrounding cells and tissues. Thus, it is very important to understand the roles of complement in tissue homeostasis and pathobiology of neural retina. Given that CS is one of many important players in multifaceted configuration of cellular, humoral, and molecular factors regulating tissue homeostasis, its dual role in protecting and harming the retina depends on the entire pathophysiology accompanying AMD development and progression. Recently, strong progress has been made towards the translation of complement-based therapeutics into the clinic. The current complement therapies aim to inhibit complement activation to control inflammation; however, the resulting concurrent loss of the “nonclassical” complement functions might be of great importance, particularly for degenerating neural retina. Therefore, the complement inhibitors that advance to the clinic need to be carefully tailored and targeted to act only in those areas where complement activation needs to be controlled.

## Figures and Tables

**Table 1:** Summary of single-nucleotide polymorphisms in genes related to complement system that are associated with increased or decreased risk of AMD development.

Complement-related gene	Position: chromosome or chromosome region	SNP identification number	Phenotype-related nucleotide substitution (if known), // *amino acid substitution* (*if known*)	Effect on AMD risk	References
C2/CFB locus	6p23.3	rs429608	G	S	[32]
CFB	6p23.3	rs4151667	TA // *L9H *	P	[34, 35]
CFB	6p23.3	rs641153	GA // *R32Q *	P	[36–40]
CFB	6p23.3	rs2072633	IVS17 (intron 17 of *CFB*)	P	[39]
C2	6p23.3	rs9332739	*E318D *	P	[34, 35]
C2	6p23.3	rs547154	IVS10 (intron 10 of *C2*)	P	[34, 39, 41, 42]
C3	19p13.3	rs2230199	CG // *R102G *	S	[43]
C7	5p	rs2329434	AT or TT	P (in CFH_402H_ homozygous individuals)	[44]
C7	5p	rs2876849	AT or TT	P (in CFH_402H_ homozygous individuals)	[44]
CFH	1q31	rs10737680	A	S	[32]
CFH	1q31	rs1061170	CC // *Y402H *	S	[43, 45–47]
CFH	1q31	rs1061170	TT // *H402Y *	P	[43, 45–47]
CFH	1q31	rs1410996	CT	S	[43]
CFH	1q31	rs800292	AA // *I62V *	P	[48]
CFH	1q31	rs1065489	TA // *D936E *	P	[49, 50]
CFH	1q31	rs3753396	AG // Q672	P	[48]
CFH	1q31	rs1329428	CFH intron	P	[51]
CFH	1q31	rs1329424	T	S	[52]
CFH	1q31	rs380390	C	S	[47]
CFH	1q31	rs10272438	C	S	[47]
CFI	4q25	rs10033900	CT	S	[43]
CFI	4q25	rs4698775	CG	S	[32]
ARMS2/HTRA1	10q26	rs10490924	GT // A69S	S	[32, 43]
ARMS2/HTRA1	10q26	rs3750848	*ARMS2* intron	S	[51]
ARMS2/HTRA1	10q26	rs3793917	HTRA1 promoter polymorphism	S	[51]
CFHR1	1q23	—	gene deletion → CFHR1 protein is absent in serum of homozygotes.	P	[53, 54]
CFHR3	1q23	—	gene deletion → CFHR3 protein is absent in serum of homozygotes.	P	[53, 54]

P: protective effect; S: increased susceptibility to AMD.
